# Comment on “*In Vitro* Effects of Bisphenol A β-D-Glucuronide (BPA-G) on Adipogenesis in Human and Murine Preadipocytes”

**DOI:** 10.1289/ehp.1510315

**Published:** 2015-12-01

**Authors:** Véronique Gayrard, Glenn Gauderat, Marlène Z. Lacroix, Catherine Viguié, Alain Bousquet-Melou, Pierre-Louis Toutain, Nicole Picard-Hagen

**Affiliations:** Institut National de la Recherche Agronomique, Université de Toulouse, Toulouse, France

Boucher et al. reported that treatment of 3T3L1 preadipocytes with 10 μM bisphenol A β-D-glucuronide (BPA-G) induced a significant increase in lipid accumulation, in mRNA expression of the adipogenic markers sterol regulatory element binding factor 1 (*SREBF1*) and lipoprotein lipase (*LPL*), and in protein levels of LPL, aP2, and adipsin. They concluded that their study was the first to show that BPA-G induced adipocyte differentiation and was not simply an inactive metabolite.

To justify the relevance of their 10-µM effective BPA-G concentration, they claimed that this tested concentration was within the range found in human fluids. To support their statement, they cited Harthé et al. (2012), who reported an average BPA-G concentration of 4.64 µg/L in human urine samples but wrongly converted this figure to a urine concentration of 11.5 µM (their effective *in vitro* concentration) rather than to the correct figure of 11.5 nM, i.e., a value 1,000 times lower.

Apart from this error, it should be stressed that the plasma concentration and not the urine concentration is the driving concentration to explain any systemic effect. As the estimated ratio of total BPA (sum of BPA and its metabolites) concentration between serum and urine was less than 0.045 (Teeguarden et al. 2013), it can be concluded that the 10-µM *in vitro* effective BPA-G concentration was several orders of magnitude higher than what would be expected to be measured in the general population.

Nevertheless, the fact that such high *in vitro* BPA-G displayed an estrogenic action merits some attention and was not totally unexpected. It should be recognized that BPA-G can be back-converted to BPA (aglycone) thanks to the ubiquitous presence of β-glucuronidases in many organs and bodily fluids (Sperker et al. 1997). Such a deconjugation process was reported to account for the estrogenic effect of soy isoflavone glucuronides on breast cancer cell lines (Islam et al. 2015). The likelihood that such a mechanism underlies the effects of BPA-G is supported by the *ex vivo* demonstration that BPA-G is readily converted back to its parent compound in ovine gonads, BPA conjugation–deconjugation cycling favoring BPA-G hydrolysis (Corbel et al. 2015). Nishikawa et al. (2010) also observed deconjugation of BPA-G in fetal liver cells, suggesting that BPA-G can enter cells and that endogenous glucuronidases may convert BPA-G to its aglycone form, about 5% of BPA-G being reactivated after 2 hours. More generally, the lack of stability of BPA-G in the presence of rodent fetal tissue homogenates has been reported (Waechter et al. 2007). Thus, it can be hypothesized that BPA-G deconjugation by the preadipocytes could result in sufficiently high residual BPA concentrations to trigger the effects reported by Boucher et al. This issue could be easily explored by analyzing the extracellular medium and the intracellular BPA content of preadipocyte cells after 48 hours of exposure.
